# CDSE-UNet: Enhancing COVID-19 CT Image Segmentation With Canny Edge Detection and Dual-Path SENet Feature Fusion

**DOI:** 10.1155/ijbi/9175473

**Published:** 2025-03-16

**Authors:** Jiao Ding, Jie Chang, Renrui Han, Li Yang

**Affiliations:** ^1^School of Electrical and Electronic Engineering, Anhui Institute of Information Technology, Wuhu, China; ^2^Department of Information, Wuhu Shengmeifu Technology Co. Ltd, Wuhu, China; ^3^School of Medical Information, Wannan Medical College, Wuhu, China

## Abstract

Accurate segmentation of COVID-19 CT images is crucial for reducing the severity and mortality rates associated with COVID-19 infections. In response to blurred boundaries and high variability characteristic of lesion areas in COVID-19 CT images, we introduce CDSE-UNet: a novel UNet-based segmentation model that integrates Canny operator edge detection and a Dual-Path SENet Feature Fusion Block (DSBlock). This model enhances the standard UNet architecture by employing the Canny operator for edge detection in sample images, paralleling this with a similar network structure for semantic feature extraction. A key innovation is the DSBlock, applied across corresponding network layers to effectively combine features from both image paths. Moreover, we have developed a Multiscale Convolution Block (MSCovBlock), replacing the standard convolution in UNet, to adapt to the varied lesion sizes and shapes. This addition not only aids in accurately classifying lesion edge pixels but also significantly improves channel differentiation and expands the capacity of the model. Our evaluations on public datasets demonstrate CDSE-UNet's superior performance over other leading models. Specifically, CDSE-UNet achieved an accuracy of 0.9929, a recall of 0.9604, a DSC of 0.9063, and an IoU of 0.8286, outperforming UNet, Attention-UNet, Trans-Unet, Swin-Unet, and Dense-UNet in these metrics.

## 1. Introduction

Corona virus disease 2019 (COVID-19), a highly contagious and severe respiratory disease, has emerged as a grave threat to global health. Since its outbreak in December 2019, COVID-19 has spread rapidly, leading to an exponential increase in infections. The World Health Organization (WHO) reports that, as of November 11, 2022, the global case count has exceeded 630 million, with the death toll surpassing 6.58 million. In this context, rapid and precise diagnostic methods are vital in curbing the high rates of severe infections and mortality associated with COVID-19. Common diagnostic tools include reverse transcription–polymerase chain reaction (RT-PCR) and reverse transcription polymerase chain. However, the propensity of RT-PCR tests to yield false negatives necessitates supplementary diagnostic measures, such as chest imaging [1]. The “Novel Coronavirus Infection Diagnosis and Treatment Plan (Trial Tenth Edition)” highlights key diagnostic indicators, including bilateral multifocal ground-glass opacities and pulmonary consolidation, observable via chest imaging, predominantly computed tomography (CT) [2]. Nonetheless, the reliability of diagnostic outcomes is often contingent on the interpreting physician's expertise, leading to potential variability in diagnoses [3–5].

Accurate segmentation of lesion areas in COVID-19 CT images is imperative in mitigating the severity and mortality of the disease. However, the pandemic's rapid spread and the sheer volume of patients render traditional manual diagnosis methods inadequate. The advent of computer technology, particularly the application of machine learning algorithms like SVM [6] and decision trees [7], initially marked advancements in medical image segmentation. Despite these developments, their efficacy remained limited. The recent surge in deep learning technologies, especially convolutional neural networks (CNNs) [8], has significantly outperformed traditional algorithms in feature extraction and robustness. Fully convolutional networks (FCNs) [9], as seminal works in image semantic segmentation, utilize upsampling in their final layer to achieve pixel-level classification. However, the simplistic upsampling approach in FCNs often results in the loss of crucial shallow semantic features, affecting segmentation accuracy, albeit still surpassing traditional algorithms.

Feature fusion can enrich feature semantics, and the attention mechanism can enhance important features. Lu et al. [10] combined condition vectors to ensure that the generated tile image has more stylistic variations. Zhu et al. [11] applied multifusion to the feature pyramid, successfully using it in the field of wood microscopic image identification. Peng et al. [12] addressing small object detection, designed a channel space mixed attention (CSMA) to enhance important detail features. 2MGAS-Net [13] significantly enhances its ability to extract detailed boundary features of colonic polyps by designing a cascaded gated attention mechanism and integrating multiscale compressed features. DermoNet [14] represents an innovative approach for detecting skin lesions, which significantly boosts the recognition performance for skin lesions by utilizing a multiscale feature layer (MSFL) module and multilevel feature fusion (MLFF) technology.

In leveraging the full potential of CNN layers' semantic features, Ronneberger et al. conceptualized the UNet model [15]. This architecture, shaped like the letter “U,” comprises 4 encoding layers, 2 bottleneck layers, and 4 decoding layers, with interconnected encoding and decoding layers. UNet structured network, requiring minimal sample training, has substantially enhanced medical image segmentation performance.

Given the important role of Unet in medical image segmentation, numerous refinements have been proposed, falling into two broad categories: structural enhancements and performance optimization. Structural enhancements generally focus on modifying encoders and decoders, as seen in variants like Res-UNet [16], Dense-UNet [17], Attention-UNet [18], Trans-UNet [19], and MltiResUNet [20]. Wang et al. [21] introduced CopleNet, a UNet-like model for segmenting COVID-19 CT image lesions, employing bridging layers and a noise-robust DSC loss function to enhance noise resilience. Feature fusion and attention mechanisms have also been successfully integrated into UNet architectures. Lu et al. [22] proposed a novel hybridoma cell segmentation method that uses multiscale feature fusion and a dual attention mechanism, demonstrating superior performance in addressing size and position variations in hybridoma cell segmentation. Additionally, UNet has been utilized for data augmentation; Li et al. [23] enhanced the accuracy of remote sensing image semantic segmentation by generating pseudolabel samples with UNet, thereby expanding the dataset.

**Figure 1:**
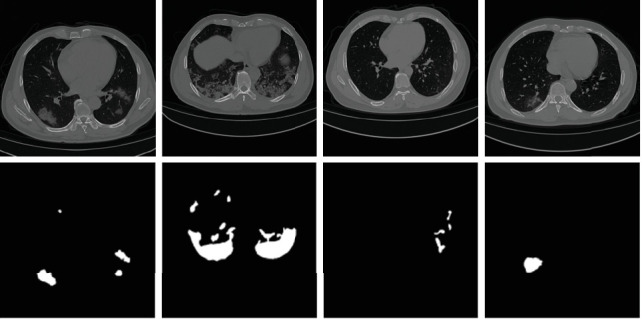
COVID-19 CT images and segmentation masks. The first row shows the original CT images of patients infected with COVID-19. The second row displays the segmentation masks that correspond to these CT images.

UNet and its evolved variants can autonomously segment lesions in COVID-19 CT images, markedly improving upon conventional methodologies. Nevertheless, challenges persist in achieving optimal segmentation accuracy and robustness. These challenges stem from the significant variability in lesion sizes and shapes, coupled with the indistinct boundaries between lesions and normal tissues in COVID-19 CT images, as illustrated in Figure 1. To address these issues, some researchers have integrated image edge detection features into CNN models to bolster edge feature extraction, thereby facilitating object edge pixel classification. Bertasius et al. [24] introduced the DeepEdge model, employing the Canny operator for initial edge information extraction, followed by semantic fusion with corresponding network features of varying sizes to enhance edge pixel classification accuracy. To tackle the diverse lesion area sizes, inspiration was drawn from the Inception network [25], leading to the design of convolutional kernels of multiple sizes to extract semantic features at different granularities. For instance, Dolz et al. [26] devised the dense multipath U-Net model to accommodate the high variability in ischemic stroke lesion characteristics, using 3 × 3, 5 × 5, and 7 × 7 convolutional kernels for multiscale semantic information extraction, thereby boosting model robustness.

**Figure 2:**
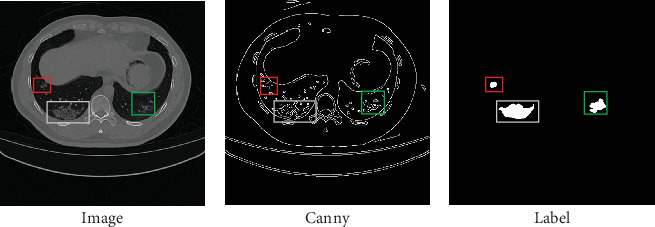
(a–c) The three images are the original image, the edge information image extracted by the Canny operator, and the corresponding lesion area image, respectively. These images exhibit a high degree of consistency between the contour features of the edges detected by the Canny operator and the contour shapes of the various lesion areas.

In addressing the challenges posed by the significant variability in lesion sizes and shapes in COVID-19 CT images, as well as the blurred boundaries between lesions and normal regions, previous researchers have primarily focused on the design and improvement of model structures, attention mechanisms, multiscale windows, and other aspects [27–30]. However, there has been limited attention given by researchers to the guiding role of lesion edge information. In this paper, we utilize the Canny edge detector to extract detailed contour edge information from the images. This information is subsequently used to guide the extraction of information related to the lung lesion areas. As shown in Figure 2, the contour features of the three edge regions in the image processed by the Canny operator are highly consistent with the three lung lesion regions in the labeled image, demonstrating that the edge information detected by the Canny operator contains important feature information about the lung lesion regions. In addition, we have designed three feature fusion methods to explore the supervisory role of edge information detected by the Canny operator.

The main contributions of this paper are as follows:

• The use of the Canny operator to generate edge detection images for samples, extracting their semantic features with the identical network structure as the samples and fusing them at corresponding network layers. This approach emphasizes edge information during training.
• The design of the DSBlock is aimed at improving channel differentiation, suppressing irrelevant features, and emphasizing relevant ones. This module is used in two scenarios: merging edge detection features with the image's inherent features and replacing skip connections between encoders and decoders.
• The development of the MSCovBlock within the encoder module, incorporating convolutional kernels of various sizes to maintain a balance between local and global semantic feature extraction.

## 2. Related Work

### 2.1. Image Segmentation Based on Deep Learning

Deep learning refers to artificial neural networks with deep structures and is currently the most popular machine learning technique. In 2012, Krizhevsky et al. introduced a deep learning algorithm called Alex Net [8], a type of CNN. In that year's ImageNet competition, it significantly outperformed traditional machine learning algorithms like SVM [6] and decision trees [7], marking the beginning of the deep learning boom. In 2015, Long et al. were the first to apply deep learning to the field of image segmentation to design FCN and achieve pixel-level image segmentation [9]. Since then, a plethora of image segmentation models based on deep learning have emerged. Lu et al. [10] enhanced tile image generation by integrating condition vectors, resulting in images with a greater variety of styles. Zhu et al. [11] employed multifusion in the feature pyramid, achieving success in the identification of wood microscopic images. Meanwhile, Peng et al. [12] tackled small object detection by designing a CSMA mechanism, which effectively highlighted essential detail features.

Ronneberger et al. designed the renowned UNet model [15]. It primarily consists of 4 encoder layers, 2 bottleneck layers, and 4 decoder layers. The network structure resembles the uppercase letter “U.” The encoder is connected to the corresponding decoder layer in the network structure. The encoder comprises convolution layers, BN (batch normalization), RELU (rectified linear unit), and downsampling. In contrast, the decoder consists of convolution layers, BN, RELU, and upsampling. The bottleneck layers contain convolution layers and RELU. Thanks to the model's encoder–decoder structure and skip connections, it can achieve semantic segmentation of images with a limited amount of training data. The model has been successfully applied to the field of medical image segmentation. Lu et al. [22] introduced an innovative method for segmenting hybridoma cells, leveraging multiscale feature fusion in combination with a dual attention mechanism. In addition, UNet has been effectively applied to data augmentation tasks, wherein Li et al. [23] enhanced the precision of semantic segmentation in remote sensing images by using UNet to generate pseudolabel samples.

### 2.2. Edge Detection

Similar to image features like shape, color, and texture, edges are among the most fundamental characteristics of an image and play a vital role in effective image analysis. An edge refers to the discontinuity in local properties of an image, such as sudden changes in grayscale or structure. Edge detection is a common digital image segmentation technique. By analyzing the differences in pixel grayscale values within an image, one can pinpoint areas of brightness variation at object boundaries, thereby extracting edge or contour information of objects within the image.

Currently, common edge detection algorithms include the Sobel operator [31], Prewitt operator [32], Roberts operator [33], and Canny operator [34]. The Roberts operator utilizes a local differential operator to detect edges in an image. While it offers good edge localization, it may result in the loss of some edges. Additionally, this operator does not include image smoothing, leading to poor noise resistance. As such, it is best suited for images with sharp edges and minimal noise. Both the Sobel operator and the Prewitt operator apply weighed smoothing to neighboring pixel points in an image, followed by a differentiation operation. However, the two operators use different weights for smoothing. They both provide decent noise resistance and good edge localization, but they might detect false contours in the edges [1]. The Canny operator uses high and low thresholds to detect strong and weak edge points in an image, respectively. Based on pre-established pixel connection rules, weak edge detection points are linked to strong edge detection points, ensuring the most accurate representation of actual object edges in the image with good edge localization. These detection operators identify the discontinuity in grayscale or gradient values of image pixels and then adjust the grayscale threshold or gradient values to detect the most prominent edges or contours of objects.

### 2.3. Channel Attention Mechanism

The attention mechanism is a technique to reinforce local information and has been extensively incorporated into various deep learning models. By adding trainable attention weights to the network model, the model can automatically adjust weight parameters during training. This enhances the weights of key regions while suppressing those of irrelevant areas, allowing the model to mimic human cognitive functions and better accomplish its tasks [35].

Attention mechanisms can be categorized into channel attention, spatial attention, and hybrid attention based on their processing of objects. Spatial attention focuses on feature map-level features, and channel attention centers on channel-level features, while hybrid attention simultaneously concentrates on both feature map and channel-level features.

Hu et al. introduced a channel attention mechanism called SENet (Squeeze-and-Excitation Networks) [36], which won the ImageNet competition that year. SENet considers the relationships between feature channels and incorporates attention mechanisms on them. SENet automatically learns the importance of each feature channel and uses the derived importance to enhance relevant features while suppressing less important ones for the current task. This is achieved through the squeeze-and-excitation modules. Algorithms improved upon SENet include ECANet (Efficient Channel Attention Network) [37], GCNet (Global Context Network) [38], and DANet (Dual Attention Network) [39]. ECANet removes the squeeze-and-excitation operations from SENet, substituting them with local cross-channel interactions, which further reduces the model's parameter count compared to SENet. GCNet introduces the global context block to obtain global information relationship vectors, providing a broader global perspective compared to SENet. DANet designs a network that merges both spatial and channel attention, with channel attention being implemented through the position attention module.

## 3. Materials and Methods

**Figure 3:**
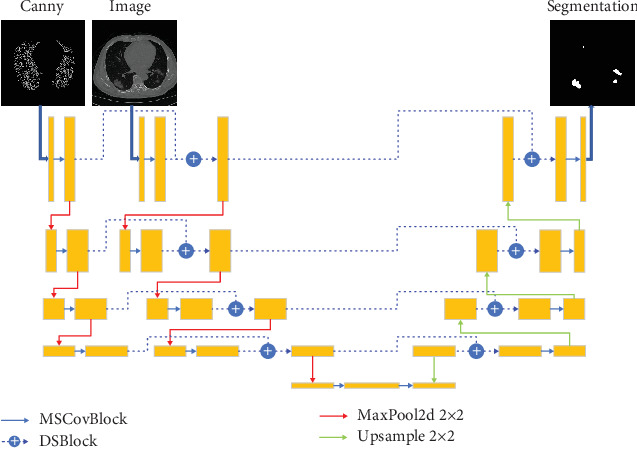
The architecture of the proposed CDSE-UNet integrates Canny edge detection, DSBlock, and MSCovBlock into a UNet framework. DSBlock as a key component enhances the feature fusion process across different network layers. MSCovBlock stands for multi-scale convolution block, which facilitates the extraction of detailed semantic features across various scales and sizes, crucial for handling the diverse lesion characteristics in COVID-19 CT images.

The CDSE-UNet model, utilizing UNet as its foundational architecture, is depicted in Figure 3. In this innovative approach, each encoder layer receives two distinct inputs: a Canny operator–based edge detection image and the original sample image. These inputs, sharing a uniform network structure, are processed using MSCovBlock, which replaces the conventional convolution in Unet, for enhanced feature extraction. The resulting features, with their channel numbers doubled yet maintaining the same window size, are fused using the DSBlock based on SENet. This fusion maintains the channel number and window size consistent. However, the origins of the two inputs differ in subsequent layers. For the sample image input, features generated from the previous layer's merged inputs through DSBlock are utilized, while the Canny edge detection image input is derived from its own convolved features. The transition between layers is achieved via pooling layers, preserving the feature channel numbers while halving the window sizes.

The model's bottleneck layer and decoder structure mirror that of the standard UNet network. The key distinction lies in the feature extraction process, conducted via MSCovBlock, and in the replacement of UNet's skip connections with DSBlock. The subsequent sections delve into the intricate details of the CDSE-UNet implementation.

### 3.1. Edge Detection Image Based on Canny Operator

Lesion areas in COVID-19 CT images, often manifesting as ground-glass opacities and infiltrative shadows, are marked by blurred boundaries with normal regions. The precision of edge features in these areas is critical for segmentation accuracy. Drawing inspiration from [24], this study integrates edge detection images into the COVID-19 CT image segmentation model to enhance the classification of edge pixels in lesion areas. Edges, akin to shape, color, and texture features, are fundamental to image analysis, representing discontinuities in local image properties like abrupt changes in grayscale or structure. Image edge detection, a prevalent technique in digital image segmentation, identifies object edge brightness variations by analyzing pixel grayscale differences. Prominent edge detection algorithms include Sobel, Prewitt, Roberts, and the pivotal Canny operators. The Canny operator, particularly, excels in detecting brightness changes through differential operations, distinguishing strong and weak edge points using high and low thresholds. This operator is known for its low edge detection error rate and superior edge positioning accuracy, outshining other operators in numerous applications.

#### 3.1.1. Fusion of Edge Detection and Sample Images

**Figure 4:**
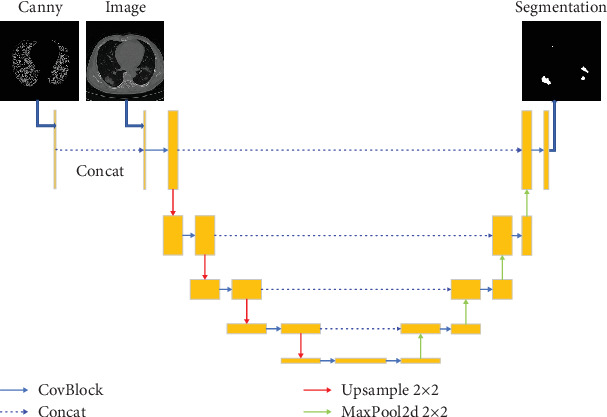
This figure demonstrates the process of feeding the CDSE-UNet model with a combined input of Canny edge-detected images and original CT scans. However, these two types of images possess complex multimodal semantic relationships, and a simple stacking approach might overlook the correlations between them, thereby diminishing the supervisory role of the edge detection image.

In image segmentation, the supervisory role of edge detection images is leveraged by directly stacking the features of the original sample image with those of the edge detection image, as visualized in Figure 4. However, these images, belonging to distinct modalities, possess complex intermodal semantic relationships as elucidated by [40]. Simple multimodal stacking could overlook these intricate correlations. Inspired by [26], which proposed multimodal image fusion paths for semantic integration at various levels, this study adopts an image feature fusion method illustrated in Figure 3. This method, treating the original sample image and edge detection image as dual inputs with identical semantic feature extraction network structures, fuses them at each network layer, thereby enhancing the correlation between the two image types and facilitating accurate classification of image edge pixels.

#### 3.1.2. Generation of Edge Detection Image Based on Canny Operator

Following the methodology outlined in [41], the Canny operator is employed to extract edge features. The process involves several steps:

1. Gaussian filtering. Gaussian filtering primarily smoothens the image, suppressing noise within it. Specifically, a two-dimensional Gaussian kernel of a particular size convolves with the image. The Gaussian kernel *G*(*x*, *y*) is presented in Equation (1).

(1) $$\begin{array}{c} G\left(x,y\right)=\frac{1}{2\pi \sigma^{2}}\exp\left(-\frac{x^{2}+y^{2}}{2\sigma^{2}}\right) \end{array}$$

2. Calculating gradient magnitude and direction for pixel points. The Sobel first-order derivative operator is used to calculate the gradient magnitude of each pixel point in the *x* and *y* directions. The Sobel operator is shown in Equation (2).

(2) $$\begin{array}{c} s_{x}=\left(\begin{array}{ccc} -1 & 0 & 1\\ -2 & 0 & 2\\ -1 & 0 & 1 \end{array}\right),s_{y}=\left(\begin{array}{ccc} 1 & 2 & 1\\ 0 & 0 & 0\\ -1 & -2 & -1 \end{array}\right) \end{array}$$

Using *S*_*x*_ and *S*_*y*_, calculating gradient matrices, *G*_*x*_ and *G*_*y*_, for grayscale image *I* in the *x* and *y* directions, respectively, as shown in Equation (3).

(3) $$\begin{array}{c} G_{x}=S_{x}*I,G_{y}=S_{y}*I \end{array}$$

The image gradient matrix *G*_xy_ is then calculated based on the formulation $g_{\text{xy}}\left(i,j\right)=\sqrt{g_{x}{\left(i,j\right)}^{2}+g_{y}{\left(i,j\right)}^{2}}$

3. Nonmaximum suppression of pixel gradient. An eight neighborhood is constructed for pixel points. Based on the sign and magnitude of gradient strengths in the *x* and *y* directions, the gradient direction region is determined. Then, using the gradient strength values of adjacent pixel points, gradient values for comparison points in the positive and negative directions are calculated using linear interpolation, as shown in Equations (4) and (5).

(4) $$\begin{array}{c} g_{\text{up}}\left(i,j\right)=\left(1-t\right)\cdot g_{\text{xy}}\left(i,j+1\right)+t\cdot g_{\text{xy}}\left(i-1,j+1\right) \end{array}$$

(5) $$\begin{array}{c} g_{\text{down}}\left(i,j\right)=\left(1-t\right)\cdot g_{\text{xy}}\left(i,j-1\right)+t\cdot g_{\text{xy}}\left(i+1,j-1\right) \end{array}$$

where *t* = |*g*_*y*_(*i*, *j*)/*g*_*x*_(*i*, *j*)|. The gradient values of the comparison points are compared with the gradient strength of the pixel point. If the gradient value of the pixel point is maximal, the pixel point is retained; otherwise, it is suppressed.

4. Threshold hysteresis. A gradient strength threshold range (*low*, *high*) is set. The retained pixel point gradient strength *k* is compared with thresholds, *low* and *high*, to determine if it should be considered an edge point in the image. There are three scenarios: (1) When *k* > high, it is retained. (2) When low ≤ *k* ≤ high, its status is pending. (3) When *k* < low, it is eliminated.
5. Elimination of isolated weak edges. Pixel points with gradient strength within the range (*low*, *high*) are marked as pending, meaning they are potential edge points that need to be checked against their eight-neighboring pixels. If any of these neighboring pixels have been retained, the pending pixel is preserved as an edge point; otherwise, it is eliminated.

### 3.2. Feature Fusion

To explore the supervisory role of edge information detected by the Canny operator, we have designed three feature fusion methods: the Simple Concatenation Block (SBlock), the Single SENet Feature Fusion Block (SSBlock), and the Double SENet Feature Fusion Block (DSBlock). For clarity and convenience, we will use these abbreviations in the subsequent descriptions.

**Figure 5:**
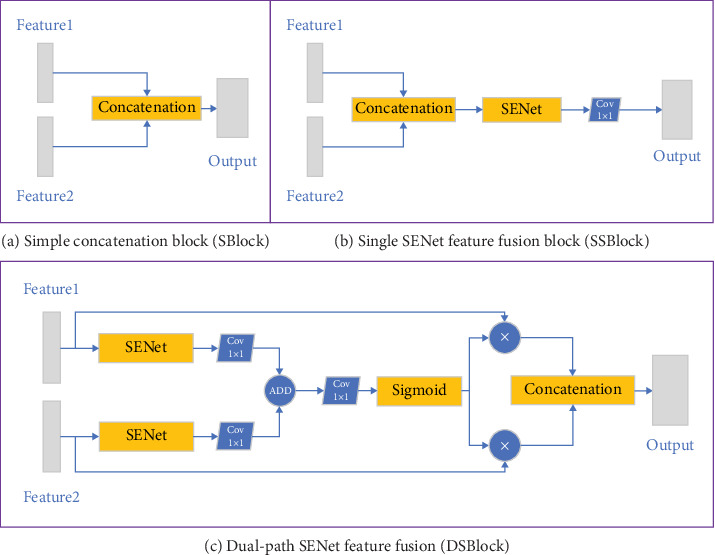
The figure presents a visual comparison of three distinct feature fusion approaches. (a) Display the SBlock, where features are straightforwardly stacked, providing a basic level of fusion. (b) Illustrate the SSBlock, which introduces the SENet mechanism for more nuanced channel-wise feature integration. (c) Depict the DSBlock, an advanced fusion approach that employs dual-path SENet structures for deeper and more effective feature integration from different image modalities.

**Figure 6:**
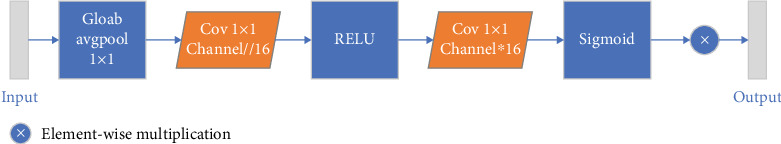
This figure illustrates the Squeeze-and-Excitation Network (SENet) architecture, which focuses on adaptively recalibrating channel-wise feature responses by explicitly modelling interdependencies between channels.

Feature fusion is initially achieved through channel stacking, as depicted in SBlock in Figure 5a. This approach treats all channels equally, yet the significance of each channel varies. The channel attention mechanism, as exemplified by SENet (Figure 6), adeptly represents channel weights, suppressing irrelevant and enhancing important features. The mechanism involves global average pooling, followed by squeeze-and-excitation operations, and finally, Sigmoid's normalization, leading to the generation of channel attention parameters which are multiplied element-wise with the input feature map to yield the output feature map.

Building on SBlock, SSBlock was introduced, as shown in Figure 5b, employing SENet to augment feature fusion efficiency. Given the distinct modalities and channel relationships of the edge detection and sample images, DSBlock was conceptualized. This block, shown in Figure 5c, first selects channel attention before merging features, thus accommodating the modalities' differences and enhancing channel differentiation, as evidenced by subsequent experimental results. In the UNet model, skip connections facilitate the addition of shallow semantic features from the encoder to the upsampled features in the decoder. Accordingly, DSBlock, a suitable replacement for these connections, was integrated into our network model.

### 3.3. Multiscale Convolution Block

COVID-19 patients typically exhibit varying lesion sizes and shapes across the incubation, progression, and recovery phases. The model's capability to segment lesions of different sizes is essential, particularly in the early detection stages to mitigate the risk of worsening conditions. The convolution kernel size in the model determines the receptive field of the input feature image. The UNet model's fixed 3 × 3 kernel size is inadequate for the diverse lesion areas seen in COVID-19. The Inception module in the InceptionNet network, comprising variously sized convolution kernels, addresses this limitation by broadening the network model.

**Figure 7:**
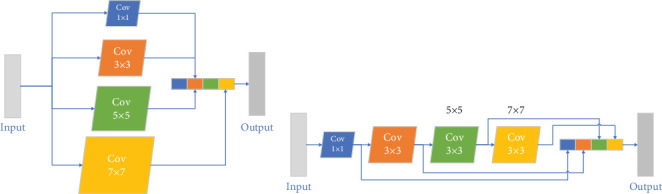
To address the diverse and complex patterns in COVID-19 CT images, the MSCovBlock consists of convolutional layers of multiple kernel sizes such as 1 × 1, 3 × 3, 5 × 5, and 7 × 7, in a parallel configuration. This setup allows the block to simultaneously process and combine information from both local and global perspectives.

This paper incorporates a multisize convolution kernel module within the network model, enhancing the extraction of both local and global lesion area features. The module includes 1 × 1, 3 × 3, 5 × 5, and 7 × 7 sized convolutions. Initially designed as depicted in Figure 7a, the model adopted in this paper, illustrated in Figure 7b, reduces computational demands while maintaining effective feature extraction.

### 3.4. Loss Function

This paper combines binary cross-entropy (BCE) and Dice Loss as the following loss function.

(6) $$\begin{array}{c} \text{Loss}=0.5*\text{BCELoss}+0.5*\text{DiceLoss} \end{array}$$

(7) $$\begin{array}{c} \text{BCELoss}=-X\log\left(Y\right)-\left(1-Y\right)\log\left(1-X\right) \end{array}$$

(8) $$\begin{array}{c} \text{DiceLoss}=1-\frac{2*\left|X\cap Y\right|}{\left|X\right|+\left|Y\right|} \end{array}$$

where *X* represents the true label for the COVID-19 CT image, while *Y* represents the corresponding predicted label.

## 4. Experiment

### 4.1. Experimental Environment

The software development environment used was PyCharm Community Edition 2021 with the development framework being PyTorch. The primary configuration environment includes python3.7, torch1.8.1, cuda111, and numpy1.20.3. The hardware environment was Nvidia GeForce GTX 3070 (8 GB memory). The primary parameters for model training include the Adam optimizer, an initial learning rate of 0.001, dynamic learning rate adjustment every 30 epochs with a decay factor of 0.9, a maximum of 300 training iterations, and retaining the model with the highest dice similarity coefficient (DSC) value on the test set as the optimal model. The implementation code for this study has been made publicly available at https://github.com/yanglichel/CDSEUnet.git.

### 4.2. Datasets

The experimental data is sourced from image slices in the public dataset, namely, the COVID-19 CT lung and infection segmentation dataset [42]. This dataset comprises 20 cases, categorized into corona cases type and pneumonia type. Experienced radiologists annotated and validated the images, with three types of annotations: left lung, right lung, and the infection area. Since most of the image slices did not display infection areas, this study selected 878 slices that predominantly featured infection areas. From these, 788 were randomly chosen as the training set and 90 for the test set. This paper focuses on segmenting the infected areas of the lungs, retaining only the infection mask data. Some data samples and their corresponding masks are shown in Figure 1.

The COVID-19 CT lung and infection segmentation dataset contains images in which the proportion of lesion areas is relatively small, accounting for only 2.12%. This results in a severe class imbalance issue within the dataset. The Dice Loss proposed in Reference [43] is effective in addressing this issue. This loss function, as shown in Formula (8), assigns a weight of 1 to lesion areas and 0 to background areas. Through *X*∩*Y* operations, the background is filtered out, enabling the model to focus more on lesion areas during training, thereby effectively mitigating the issue of imbalanced image data. However, when the foreground objects are excessively small, their size may lead to nonconvergence of the Dice Loss. Therefore, we combine Dice Loss with BCELoss to formulate our loss function, as detailed in Formula (6).

### 4.3. Evaluation Metrics

This study employs five commonly used image segmentation metrics for quantitative assessments: accuracy, precision, recall, DSC, and intersection over union (IoU). Accuracy evaluates the correctness of predicted pixel points. Precision assesses the accuracy of pixels predicted as positive samples. Recall measures the proportion of correctly predicted positive samples out of all positive samples. The DSC and IoU are used to evaluate the similarity and overlap between predicted results and ground truth labels, respectively. All metrics range between 0 and 1, with performance being better as they approach value 1.

## 5. Discussion

### 5.1. Results

This study compared the lung CT image segmentation network, CDSE-UNet, proposed in this paper with advanced medical image segmentation models such as UNet, Attention-UNet, Swin-Unet [44], Trans-UNet, and Dense-UNet. All experiments were conducted under the same hardware environment, and training parameters like learning rate, batch size, loss function, max pooling, and upsampling were kept consistent across all models. The number of epochs was uniformly set to 300 for all models, and the highest DSC value on the test set was chosen as the experimental result for each model. Detailed metrics for each experiment are presented in Table 1.

Table 1 shows that the CDSE-UNet model achieves the best values in terms of accuracy, recall, and DSC metrics, especially showing significant advantages in the recall and DSC metrics. In terms of the DSC metric, CDSE-UNet reaches 90.63%, making it the only model exceeding 90%, and shows an improvement of 1.88% compared to the second-best model, Dense-UNet. For the recall metric, CDSE-UNet improves by 0.84% compared to the next best model, Attention-UNet. In the accuracy metric, given that this task is a binary classification with background pixels easily classified correctly and substantially outnumbering infection area pixels, all models score high with minimal differences. Nevertheless, CDSE-UNet still performs the best, surpassing the next best model, Attention-UNet, by 0.06%. However, in the precision metric, CDSE-UNet falls short by 0.7% compared to the best-performing model, Attention-UNet. CDSE-UNet exhibits a significant advantage in the crucial DSC metric, demonstrating the strongest ability in precise segmentation of lesions.

To determine the statistical significance of the differences in performance metrics (accuracy, precision, recall, and DSC) between CDSE-UNet and the other models, each model is conducted five times, and we performed paired *t*-tests for each metric. The *p* values obtained from these tests are shown in Table 2.

### 5.2. Comparison of the Proposed CDSE-UNet With Eight State-of-the-Art Methods for COVID-19 CT Segmentation

We conducted an extensive investigation into various COVID-19 CT image segmentation methods to provide a more thorough evaluation of the performance of our novel method. As illustrated in Table 3, when compared to the current state-of-the-art techniques for COVID-19 CT segmentation, our proposed CDSE-UNet exhibits excellent segmentation performance. Specifically, its segmentation performance surpasses Inf-Net [27], Improved DeepLabV3+ [28], ACC-UNet [29], AISNetx [30], LDE-UNet [46], and PRAU-Net [48] and is roughly equivalent to that of SER-UNet [47] and DNAS-Net [49]. This is attributed to its innovative integration of the Canny edge detection, DSBlock, MSCovBlock, and a distinctive model architecture.

### 5.3. Visual Analysis

**Figure 8:**
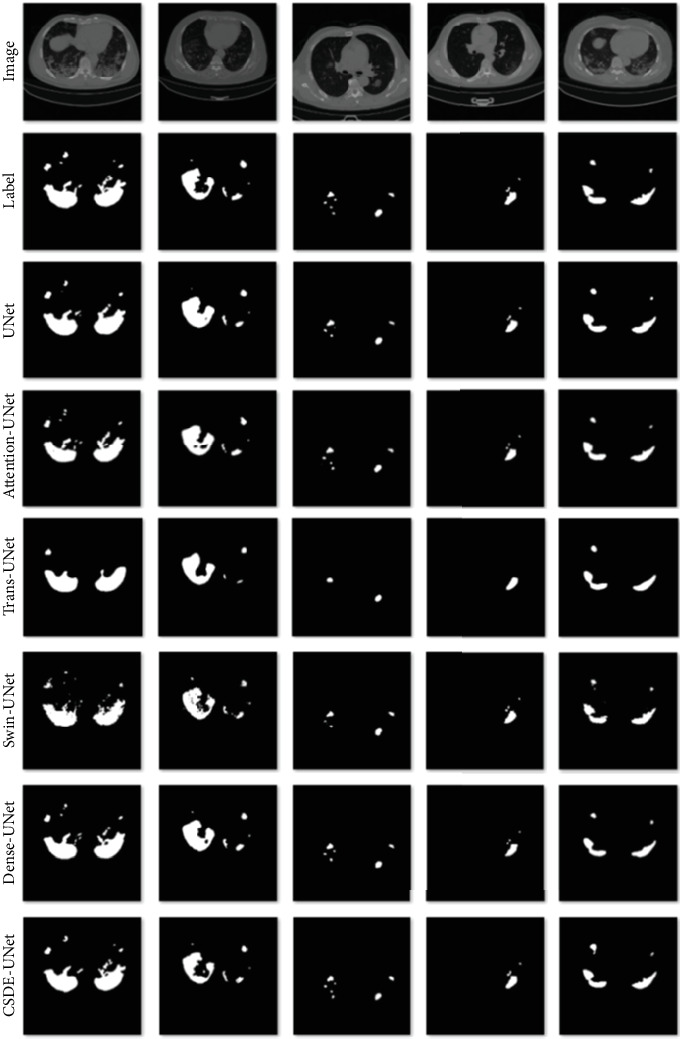
The segmentation comparison of CDSE-UNet with other counterpart models on five CT images.

Figure 8 shows the segmentation comparison of CDSE-UNet with other advanced models on five CT images. The images corresponding to “image” are the original images, those corresponding to “label” are the segmentation labels, and the images corresponding to the model names represent the segmentation results of each model. CDSE-UNet has obvious advantages in segmenting large lesion areas, small lesion areas, and lesion edges and in suppressing noise points. In the first and second images from the left in the figure, the lesion areas vary in size, and the lesions segmented by CDSE-UNet are the closest to the Label image. In the third image from the left, there are five lesions, one large and four small, in the left lung. Only CDSE-UNet segmented all five lesions, and the lesions segmented by CDSE-UNet have shapes that are more similar to the Label image. In the fourth and fifth images from the left, the lesion edges segmented by CDSE-UNet are closer to the label image.

The effectiveness of the Canny operator in edge detection is particularly notable for larger lesions, where precise edge definition is crucial. By capturing finer edge details, the Canny operator allows CDSE-UNet to delineate the boundaries of large lesions more accurately, ensuring that significant regions are not missed during segmentation. This is visually apparent in the first two images from the left of Figure 8, where the edges of the lesions closely match the ground truth labels.

Moreover, the integration of DSBlock and MSCovBlock addresses the variability in lesion sizes and enhances the model's ability to capture detailed features across different scales. The MSCovBlock allows the model to process features at various granular levels, making it adept at identifying both large and small lesion areas. The DSBlock further refines this process by selectively emphasizing important features while suppressing irrelevant ones. In the third, fourth, and fifth images from the left in Figure 8, this enhanced capability is evident as CDSE-UNet successfully segments small lesion areas that are often challenging for other models. The precise segmentation of these smaller lesions highlights the robustness and versatility of CDSE-UNet in handling diverse lesion sizes and shapes, ultimately contributing to more accurate and reliable medical image analysis.

Overall, the combination of Canny edge detection, DSBlock, and MSCovBlock ensures that CDSE-UNet not only excels in segmenting large lesion areas but also maintains high performance in detecting and accurately outlining smaller lesions, thus providing comprehensive and detailed segmentation results.

### 5.4. Ablation Experiments

#### 5.4.1. Comparative Experiment of Different Edge Detection Operators

**Figure 9:**
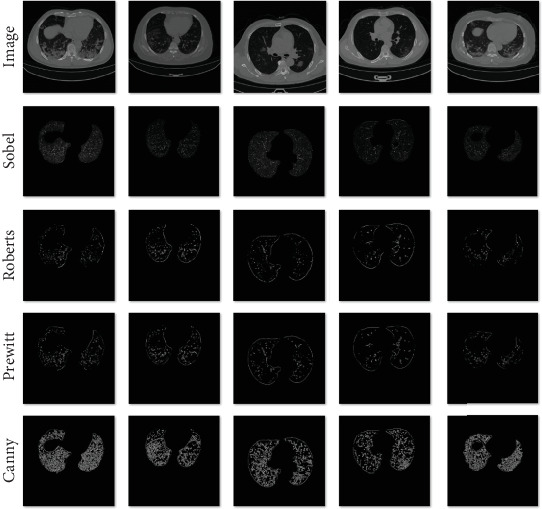
The comparison of four models in the ablation experiment.

The edges of an image contain a wealth of feature information, and edge detection operators are used to extract this edge information. Inspired by Swin-UNet, this study uses various edge detection algorithms to process sample data, generating sample edge images. These images are merged with the samples via channel connections and fed into the model for training. This approach makes the model focus more on edge features, which can enhance the performance of image segmentation. Different edge detection operators perform differently in various scenarios. To find an operator suitable for segmenting the edges of infection areas in COVID-19 CT images, we compared the Prewitt, Roberts, Sobel, and Canny edge detection operators.  Figure 9 shows the results of these four operators on five COVID-19 CT images, with a graythresh value of 0.2. From Figure 9, it can be observed that, compared to the other three operators, the Canny operator detects more image edge details, and its edges are also smoother.

Table 4 compares the evaluation metrics of the CDSE-UNet network combined with each of the four edge detection operators. The combination of the Canny operator with CDSE-UNet outperforms the combinations of other operators with CDSE-UNet in terms of accuracy, recall, and DSC metrics.

#### 5.4.2. Comparative Experiment on Different Feature Fusion Methods

In Section 3.2, we described three different methods of channel feature fusion, which are used to merge the semantic features of image edge detection, the image's own semantic features, and the skip connections between the encoder and decoder. These three methods are SBlock, SSBlock, and DSBlock, corresponding to the network structures shown in Figures 5a, 5b, and 5c, respectively. To validate the effectiveness of different feature fusion methods, an ablation study was conducted, and the results are shown in Table 5. As can be seen from the table, the third method, DSBlock, achieved the highest values in accuracy, precision, recall, and DSC, confirming the effectiveness of this feature fusion approach.

**Figure 10:**
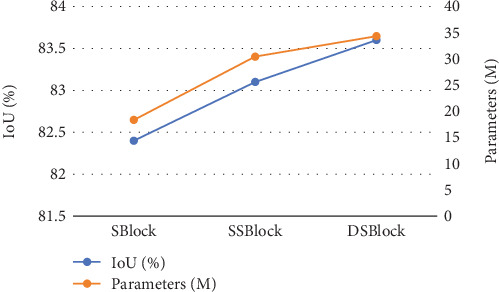
Comparison trend chart of parameters and IoU for different feature fusion methods.

In Table 5, there are notable differences in the parameters associated with the three feature fusion methods, whereas the performance variations among them are relatively minor. To further investigate the relationship between model performance and model complexity under different feature fusion methods, Figure 10 utilizes a line chart to illustrate in detail the trends in parameters versus IoU for various feature fusion methods. When using the SBlock, the model exhibits the lowest complexity, with parameters amounting to only 18.36 M, and its IoU value is 82.4%. Compared to the SBlock, the SSBlock substantially increases the model's complexity, with its parameters reaching 30.44 M, almost doubling, yet the IoU improves by only 0.7%. Compared to the SSBlock, the DSBlock results in an increase in parameters by about one-tenth, while the IoU value further increases by 0.5%. Notably, the proportional increase in the IoU value surpasses that of the parameters.

Evidently, different feature fusion methods exert a more pronounced impact on model complexity than on model performance. Although complex feature fusion methods can indeed improve the segmentation accuracy of the model, they also introduce considerable computational overhead. The relationship between model complexity and model performance is not a simple linear increase. Rather, model performance is dependent on the rational design of feature fusion methods. This is evident from the comparison between the two complex feature fusion methods: The DSBlock has slightly more parameters than the SSBlock, but its performance improvement is notably more significant. This information provides valuable insights for evaluating and enhancing model performance.

## 6. Conclusion

This study presents CDSE-UNet, an innovative deep learning model specifically designed for segmenting COVID-19 CT images. The CDSE-UNet model seamlessly integrates sample edge detection features using the Canny operator with a fusion method named DSBlock. The Canny-based feature allows the model to focus more intently on lesion edge pixels, while the DSBlock enhances the discriminatory power of channel features and improves the representation of vital features. Furthermore, the model also incorporates the MSCovBlock, effectively balancing the extraction of local and global features. Extensive comparative experiments, conducted on benchmark datasets, have revealed that CDSE-UNet exhibits outstanding segmentation performance, with an accuracy of 0.9929, a recall of 0.9604, a DSC of 0.9063, and an IoU of 0.8286. This sufficiently demonstrates that CDSE-UNet has the potential to substantially improve traditional COVID-19 CT image segmentation methodologies. The model holds the promise of providing doctors with quantitative diagnostic insights, thereby reducing diagnostic timeframes and enhancing diagnostic precision. However, numerous edge detection methods exist, and this study has so far only compared four such operators—Prewitt, Roberts, Sobel, and Canny. Future research will explore more edge detection operators and their improvements to further enhance segmentation performance.

## Figures and Tables

**Table 1:** Comparison of evaluation metrics for different methods (%).

**Method**	**Accuracy**	**Precision**	**Recall**	**DSC**	**IoU**
UNet [15]	99.23 ± 0.021	77.74 ± 0.347	92.41 ± 1.002	88.20 ± 0.959	78.90 ± 1.532
Attention-UNet [18]	99.23 ± 0.018	81.40 ± 0.998	95.20 ± 0.489	87.96 ± 0.974	78.52 ± 1.545
Trans-Unet [19]	99.15 ± 0.016	80.38 ± 0.778	89.41 ± 0.653	85.59 ± 0.875	74.82 ± 1.335
Swin-Unet [44]	99.16 ± 0.019	79.19 ± 0.596	91.39 ± 0.842	86.65 ± 0.755	76.45 ± 1.167
Dense-UNet [17]	99.16 ± 0.023	81.35 ± 0.676	93.03 ± 1.252	88.75 ± 0.703	79.78 ± 1.134
CDSE-UNet	99.29 ± 0.009	80.70 ± 0.631	96.04 ± 0.595	90.63 ± 0.521	82.86 ± 0.868

**Table 2:** Paired *t*-tests in four metrics between CDSE-UNet and the other models.

**Comparison**	**Accuracy**	**Precision**	**Recall**	**DSC**	**IoU**
CDSE-UNet vs. UNet	< 0.01	< 0.01	< 0.01	< 0.01	< 0.01
CDSE-UNet vs. Attention-UNet	< 0.01	> 0.05	< 0.05	< 0.01	< 0.01
CDSE-UNet vs. Trans-Unet	< 0.01	> 0.05	< 0.01	< 0.01	< 0.01
CDSE-UNet vs. Swin-Unet	< 0.01	< 0.01	< 0.01	< 0.01	< 0.01
CDSE-UNet vs. Dense-UNet	< 0.01	> 0.05	< 0.01	< 0.01	< 0.01

*Note:* The results indicate that the differences in performance metrics that excluded precision between CDSE-UNet and the other models are statistically significant, with *p* values less than 0.05 in most comparisons and even less than 0.01 in several key metrics.

**Table 3:** Comparison of the proposed CDSE-UNet with eight state-of-the-art methods for COVID-19 CT segmentation.

**References**	**Models**	**Datasets**	**Results**
Fan et al. [27]	Inf-Net	COVID-19 CT lung and infection segmentation dataset	DSC = 59.7
Wang et al. [28]	Improved DeepLabV3+	COVID-19 CT lung and infection segmentation dataset	IoU = 82.56
Ibtehaz and Kihara [29]	ACC-UNet	COVID-19 CT lung and infection segmentation dataset	DSC = 73.99
Bai et al. [30]	AISNet	CC-CCII segmentation dataset [45]	DSC = 80.00
Wang et al. [46]	LDE-UNet	COVID-19 CT lung and infection segmentation dataset	DSC = 80.20
Jiang et al. [47]	SER-UNet	COVID-19 CT lung and infection segmentation dataset	DSC = 90.88
Zeng and Cui [48]	PRAU-Net	COVID-19 CT lung and infection segmentation dataset; CC-CCII segmentation dataset	DSC = 83.64
Liu et al. [49]	DNAS-Net	COVID-19 CT lung and infection segmentation dataset	IoU = 82.8

**Table 4:** Comparison of evaluation metrics among (percent).

**Method**	**Accuracy**	**Precision**	**Recall**	**DSC**	**IoU**
Sobel & CDSE-UNet	99.28	82.71	94.83	90.62	82.85
Roberts & CDSE-UNet	99.27	80.16	94.21	89.99	81.80
Prewitt & CDSE-UNet	99.28	82.37	96.21	90.71	83.00
Canny & CDSE-UNet	99.30	81.46	96.48	91.07	83.60

**Table 5:** Comparison of three different feature fusion methods.

**Feature fusion method**	**Accuracy**	**Precision**	**Recall**	**DSC**	**IoU**	**Parameters**
SBlock	99.27%	80.37%	96.17%	90.35%	82.40%	18.36 M
SSBlock	99.28%	80.85%	96.29%	90.77%	83.10%	30.44 M
DSBlock	99.30%	81.46%	96.48%	91.07%	83.60%	34.35 M

## Data Availability

The partial data used to support the findings of this study are included in the article, and other data used to support the findings of this study are available on request from the first author.

## Nomenclature

BCE: binary cross-entropy
BN: batch normalization
CDSE-UNet: enhancing COVID-19 CT image segmentation with Canny edge detection and Dual-Path SENet Feature Fusion
CNN: convolutional neural network
DSC: dice similarity coefficient
DSBlock: Dual-Path SENet Feature Fusion Block
FCN: fully convolutional networks
MSCovBlock: Multiscale Convolution Block
ReLU: rectified linear unit
SBlock: Simple Concatenation Block
SENet: Squeeze-and-Extend Networks
SSBlock: Single SENet Feature Fusion Block
SVM: support vector machine
UNet: neural networks with U-Net architecture for image segmentation
